# Physician advisor rotation—Filling a gap in resident education

**DOI:** 10.1002/jhm.13575

**Published:** 2024-12-15

**Authors:** Bina Patel, James Kelly, Susan Fisher, Christopher Boyle

**Affiliations:** ^1^ Physician Advisor Services Endeavor Health Evanston IL USA; ^2^ Department of Medicine Endeavor Health Evanston IL USA; ^3^ Outcomes Research Network, Research Institute Endeavor Health Evanston IL USA

Physician advisors act as liaisons between hospital administrators and clinicians, providing guidance on compliance, documentation requirements, care coordination, discharge planning, hospital stewardship, and the quality, safety, and efficiency of care delivery.^1^ They offer trainees insights into improving care quality, safety, and value while helping them avoid delays, uncoordinated care transitions, and inefficiencies in patient throughput, all while ensuring adherence to payor and regulatory standards.^2^ These skills align with the Accreditation Council for Graduate Medical Education (ACGME) milestones of patient‐centered care, physician roles in the healthcare system, and systems‐based quality improvement (QI) and patient safety education.^3^, ^4^ While QI and patient safety training is widespread, a physician advisor curriculum for residents has not been described in the literature. This perspective describes our experience developing and implementing a physician advisor rotation, as well as lessons learned.

## GOALS AND CURRICULUM DESIGN

Our goal is to introduce residents to the role of a physician advisor, focusing on quality, efficiency, safety, and care delivery. The idea for this elective arose during the onboarding of new physician advisors. Several noted that they wished they had been exposed to this specialty and educated on its importance during their medical training. Others mentioned that their work as physician advisors made their clinical duties less stressful—they faced fewer documentation queries, knew how to admit patients to the correct admission status, and were better equipped for difficult conversations with patients and families around clinical appropriateness of discharging to a skilled nursing facility (SNF).

This gap in medical education inspired the creation of a physician advisor rotation. At the time, the Internal Medicine Residency at NorthShore University Health System, now part of Endeavor Health, lacked a formal curriculum for learners in the fields of quality improvement and patient safety and physician advisor services.

**Figure 1:**
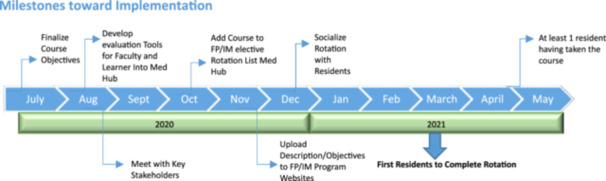
Timeline after creating a novel physician advisor and QI and patient safety rotation and milestones toward implementation.

The course directors, who serve as both physician advisors and hospitalists, collaborated with curriculum development leaders from the University of Chicago Graduate Medical Education (GME) program to define the rotation's learning activities and create an evaluation plan. Learning activities are outlined in Table 1. Both the Internal Medicine and Family Medicine residency programs expressed interest in implementing a physician advisor rotation. Funding is provided by the Departments of Medicine and Quality and Care Transformation, supporting physician advisors' salaries and administrative needs. A description of the rotation is distributed to residents before the next academic year's schedule request deadline to give them time to consider this elective. A timeline outlining the rotation's implementation is included in Figure 1.

**Table 1:** Learning activities.

Learning activities
Name 5 responsibilities of a Physician Advisor. Complete 5 Utilization Management reviews. Complete 5 Peer to Peer discussions with a medical director of a payor. Describe the 2‐midnight rule for Medicare. Describe the intensity of service and severity of illness for commercial payors. Describe 1 impact of a denial from a patient and hospital perspective. Complete 5 chart audits to identify documentation improvement opportunities. Explain why the full picture of clinical reasoning in documentation is important in care delivery. Describe the difference between facility and professional billing and define terms including case mix index (CMI), diagnosis‐related group (DRG), comorbid condition/major comorbid condition (CC/MCC), and observed to expected (O:E) ratio. Identify 2 responsibilities of a hospital care manager. Identify 5 quality leaders and interview them on their roles in the corporation. Identify one patient safety issue in the hospital and understand the purpose of the event reporting system. Complete 10 modules from Institute for Healthcare Improvement (IHI) and Dell School of Medicine on QI and patient safety, meet all objectives for each course, and receive individualized coaching in QI methods.

## STRUCTURE

Post‐Graduate Year (PGY) two and three residents participate in a 2‐week rotation, meeting in faculty offices during elective time. At the start of the rotation, the schedule, goals, and objectives are reviewed. Residents complete a pretest and posttest adapted from a published quality and patient safety curriculum,^5^ but with added sections covering the scope of physician advisor practice and local leadership identification (Supporting Information S1: Figure 1).

Learners are responsible for self‐directed study, which includes reading *The Physician Advisor Handbook* ^2^ and completing web‐based modules. The quality improvement and patient safety education is accomplished through modules from The Institute for Healthcare Improvement (IHI) Open School and Dell School of Medicine. Residents gain daily experiential learning through direct mentorship with a physician advisor, covering Utilization Management (UM) reviews, peer‐to‐peer discussions with medical directors of payors, clinical documentation integrity (CDI) activities, and appropriate use of SNFs.

Participation in quality meetings is required, including multidisciplinary huddles with case managers and social workers, hospital and surgical quality conferences, and root cause analysis (RCA) sessions. Residents also meet with at least seven quality experts to review QI and patient safety web‐based modules and complete a mock improvement exercise using the Model for Improvement. Daily debriefs with course directors allow for reflection and feedback. (A sample schedule is provided in Supporting Information S1: Figure 2).

## INITIAL OUTCOMES

From 2021 to 2023, 11 residents (34% of the PGY 2 and PGY 3 cohorts) completed the 2‐week physician advisor and quality improvement and patient safety rotation. The average pretest score was 53%, while the posttest score was 73% (*p* = <.001; Supporting Information S1: Figure 1 for test questions). Ninety percent of learners found the content was “just the right amount” and “the balance between the physician advisor and quality improvement and patient safety portion was good.” Every learner reported that the curriculum enhanced their knowledge of physician advisor services and QI and patient safety, as well as on how to effectively follow‐up on event reports.

Learners also reported an increased appreciation for the importance of physician advisors and found hospital leadership more accessible. Representative qualitative comments related to the elective included:

- “How can I moonlight as a physician advisor in my 3rd year of residency?”
- “Working with the physician advisor was great, and I really got an idea/understanding of what they do. It was a good balance between that and quality improvement and patient safety.”
- “…So nice that leaders made themselves accessible to me. The online modules and the individualized meetings afterwards were great to have.”
- “An event report is not punitive which is what I thought, but I learned about Just Culture and the purpose of event reports.”

Based on the qualitative and quantitative feedback, initial goals to provide residents an introduction to physician advisor work and quality improvement and patient safety training were met.

## LESSONS LEARNED

As residents completed the rotation, their feedback was incorporated for iterative improvements. For example, one learner said, “I still don't know the exceptions to the 2 Midnight Medicare rule,” prompting faculty to dedicate more time to Medicare inpatient admission criteria. Another requested, “More time at the beginning of the rotation reviewing modules is needed,” leading to a schedule adjustment, allowing residents to complete self‐directed learning on their first day. Feedback such as, “Some of the physician advisor modules were repetitive,” resulted in efforts to reduce redundancy. A learner suggested, “Use the QI portion as a jumpstart to a resident's QI project,” prompting course directors to focus on early QI project identification, ensuring residents could apply quality improvement tools learned during the rotation while receiving mentorship and feedback.

## FUTURE OPPORTUNITIES

To understand the impact of a physician advisor elective, outcome measurements beyond resident satisfaction and knowledge acquisition is needed. Qualitative feedback indicated that the rotation prepared residents for clinical practice by covering healthcare finance topics not typically addressed elsewhere.

Future outcomes that could be measured from this rotation include changes to physician practice, such as improved accuracy in determining correct admission status and reduced documentation queries, potentially leading to reduced patient costs, enhanced reimbursement, and appropriate length of stay. Similarly, measuring a learner's ability to overturn inpatient or SNF denials can translate to recovered revenue or reduced out‐of‐pocket patient expenses. By equipping residents with a new skillset to reduce documentation burden, physician stress or physician satisfaction can be measured for future learners. Bridging the gap between learners' clinical documentation improvement and accurate coding can significantly impact a hospital's mortality and length‐of‐stay index, making the the return of investment in training the next generation of practicing physicians measurable. This is how physician advisors have shown their value to hospital systems.

When residents attend reviews of long length‐of‐stay or outlier cases, they witness a collaborative, system‐based approach between physician advisors and hospitalists focused on patient throughput. By participating in a process that is often invisible to them and interacting with individuals whose roles they may not typically encounter, or even know exist, residents gain a new appreciation for the complex teamwork required to deliver care in a hospital setting. Although difficult to measure, this can have a profound impact in the way a resident manages challenging cases related to discharge planning. One former resident, now a hospitalist, remarked, “I haven't learned this much new information since medical school ended, as residency has been building on what I already learned.”

## CONCLUSION

Much of a physicians' work occurs outside of direct patient care. Teaching residents about physicians' roles within the healthcare system is an ACGME milestone,^4^ but is not explicitly covered in many residency programs. Our rotation provides a comprehensive introduction to physician advisor work, including admission status determination, effective advocacy during payor peer‐to‐peer discussions, clinical documentation as a narrative of care, and quality improvement and patient safety training to improve high‐value care system‐wide.

Using learner feedback, the rotation will continue to evolve to meet these objectives. A physician advisor rotation holds significant value for healthcare organizations and individual clinicians seeking to improve compliance, documentation, care coordination, discharge planning, hospital stewardship, and quality, safety, and efficiency of care delivery.

## CONFLICT OF INTEREST STATEMENT

The authors declare no conflict of interest.

## Supporting information

Supporting information.

## Data Availability

The data that support the findings of this study are available upon reasonable request from the corresponding author [BP] and non‐digital data supporting this study are available at Evanston Hospital. This GME rotation received funding by the Departments of Medicine and Quality and Care Transformation at NorthShore University Health System.
