# Supplementary material for: Physician advisor rotation—Filling a gap in resident education
Source: J Hosp Med. 2024 Dec 15;20(7):776–9. doi: 10.1002/jhm.13575 (PMC12217421; doi:10.1002/jhm.13575)
Supplement: Supplementary file 1 — Supporting information. [file JHM-20-776-s001.docx]

**Supporting Figure 1: Sample Schedule for Physician Advisor and QI and Patient Safety Rotation**

| **Date/Time** | **Topic** | **Notes** |
| --- | --- | --- |
| 7:00 - 8:00 | Surgical Quality Meeting | Virtual |
| 8:00 - 9:00 | Grand Rounds |  |
| 9:00 - 12:00 | Hierarchical Condition Coding (HCC) | Physician Advisor office |
| 10:30 - 11:30 | Congregate Living Review Committee | Conference Call |
| 12:00 - 12:30 | Lunch |  |
| 12:30 - 1:00 | Office Hours/Daily Debrief | Conference Call |
| 1:00 - 2:00 | QIPS |  |
| 2:00 - 3:00 | Quality & Patient Safety Discussion | In person |

**Supporting Figure 2: Pre and Posttest Provided to Learners**

Physician Advisor and QI and Patient Safety Rotation Pre and Posttest Adapted Version**^5^**

Name:

1. Current Year in Training during (circle one): PGY-I PGY-II

PGY- III PGY-IV

Other: ___________

1. Residency area
   1. Medicine
   2. Surgery
   3. Pediatrics
   4. Family medicine
   5. Med/Peds
   6. Other
2. Overall how much do you agree with the following statements …. ? (Please circle one response)

|  | **Strongly Agree** | **Somewhat Agree** | **Neither Agree**  **nor Disagree** | **Somewhat Disagree** | **Strongly Disagree** |
| --- | --- | --- | --- | --- | --- |
| **… I believe quality improvement knowledge is important to my education** | 5 | 4 | 3 | 2 | 1 |
| **… I believe patient safety knowledge is important to my education** | 5 | 4 | 3 | 2 | 1 |
| **… I believe learning about what a physician advisor does is important to my education** | 5 | 4 | 3 | 2 | 1 |
| **… I can identify the hospital leaders at NorthShore University HealthSystem (NSUHS)** | 5 | 4 | 3 | 2 | 1 |
| **… I believe the hospital leaders at NSUHS are accessible** | 5 | 4 | 3 | 2 | 1 |
| **… I have filed a report when my patients experienced an adverse event** | 5 | 4 | 3 | 2 | 1 |
| **… I have been part of an event report filed by another team member ( i.e. RN or resident on my team)** | 5 | 4 | 3 | 2 | 1 |
| **… I understand how to effectively follow up on event reports** | 5 | 4 | 3 | 2 | 1 |
| **… I am confident in my ability to review a Quality Dashboard to identify an area requiring improvement** | 5 | 4 | 3 | 2 | 1 |
| **… This curriculum improved my knowledge of patient safety/quality improvement at NSUHS** | 5 | 4 | 3 | 2 | 1 |

1. Which of the following patient safety event reports would require NorthShore University HealthSystem to conduct a Root Cause Analysis (RCA)? ( Circle correct answer)

- - 1. A 67 year old patient admitted for GI bleeding and anemia falls on the way to the bathroom and breaks her hip. The patient recovers well.
    2. A medical alarm on a telemetry monitor fails to activate during ventricular tachycardia event, the nurse is busy with another sick patient does not check on the patient, the patient dies.
    3. An unwrapped endotracheal tube within a code cart is used in the treatment of a patient who has stopped breathing in the MICU.
    4. A type I diabetic adolescent patient presents in the ED with glucose spiked dangerously high. The patient is given an improper dosage of insulin and experiences a severe episode of hypoglycemia and is admitted to the MICU.
    5. A patient presents to ER with abdominal pain, an abdominal aneurysm is identified, patient requires 10 units of packed red blood cells and dies on the operating table
  1. What does the Quality, Safety, Risk team do when a significant active failure is identified in a safety event report? ( Circle correct answer)
     1. Report the event to the Joint Commission
     2. Conduct a Root Cause Analysis
     3. Contact the care team involved in the incident report for more information
     4. Perform a Failure Modes and Effects Analysis
  2. A 21-year-old college student with an only documented penicillin allergy is given a tetracycline for the first time for an episode of Chlamydia. He develops a rash from the medication. This incident is best described as (Circle correct answer)
     1. a potential adverse drug event
     2. a preventable adverse drug event
     3. a non-preventable adverse drug event.
     4. a latent error.
  3. A root cause analysis meeting. ( Circle correct answer)
     1. addresses the list of event reports submitted each month
     2. is a prospective method for detecting safety hazards
     3. uses the systems approach to identify both active errors and latent errors
     4. finds the main cause of a patient safety event
     5. is mandated by the Joint Commission to analyze all patient safety events
  4. How many patient safety events have you been involved in reporting in the past 3 months? (Fill in the blank)
  5. The Institute for Medicine (IOM) identified 6 core aims for improvement. These aims say that health care should be…
     1. Straightforward, Trusted, Easy, Effective, Efficient, Patient Centered
     2. Straightforward, Timely, Effective, Accessible, Equitable, Patient Centered
     3. Straightforward , Timely, Easy, Efficient, Equitable, Patient Centered
     4. Safe, Trusted, Effective, Efficient, Accessible, Patient Centered
     5. Safe, Timely, Effective, Efficient. Equitable, Patient Centered
  6. Which of the following is true regarding the Hospital Compare website? (Circle correct answer)
     1. It provides reviews of doctors
     2. Compares quality performance for hospitals
     3. Compares liability awards between hospitals
     4. Compares complication rates for doctors
  7. Internal quality & performance measures at NorthShore University HealthSystem like the Acute Care Scorecard (choose correct answer)
     1. Only used for malpractice defense and with legal's approval
     2. Highly confidential and reported only to the Board of Trustees and senior leadership
     3. available to select individuals with a need to know
     4. available to any clinician who receives special approval
     5. available to all employees and clinicians via the intranet (Pulse)
     6. posted on the main NorthShore website (www.northshore.org)
  8. Label the following Quality Measures as Structure, Process or Outcome Measures (1=Structure, 2=Process, 3=Outcome)
     1. Percent of patients who receive flu vaccine ______
     2. Percent of kids with asthma who receive regular care who present to ER ______
     3. 30 day re-admission rate ______
     4. Number of CT scanners in a hospital ______
     5. Surgery patients whose preventive antibiotics were stopped at the right time ______
     6. Patient satisfaction scores ______
  9. For FY2023 NorthShore University HealthSystem Acute Care Scorecard includes the following measures **EXCEPT:** (Circle correct answer)
     1. Mortality – All Cause (O:E)
     2. Percent of patients discharged to a skilled nursing facility (SNF)
     3. Early Discharges
     4. 30-day hospital readmissions
     5. Sepsis Bundle Compliance
  10. In the Institute for Healthcare Improvement model of improvement “PDSA” stands for (Circle correct answer):
      1. Prepare, Develop, Study, Assess
      2. Prepare, Do, Standardize, Assess
      3. Plan, Do, Study, Act
      4. Plan, Develop, Simplify, Act
      5. Plan, Develop, Simplify, Assess
  11. A well written AIM statement should be (Circle correct answer):
      1. Targeted
      2. Measurable
      3. Simple
      4. Generalizable
  12. Name Two responsibilities of a care manager
      1. ­­­
  13. In which circumstance, would inpatient level of care be correct for a Medicare beneficiary?
      1. There is a delay in delivery of hospital care (stress test, MRI brain, etc..) so inpatient status is acceptable as the patient will cross 2 midnights completing work-up.
      2. The extended stay beyond 2 midnights is for social reasons or convenience so inpatient status is acceptable.
      3. Ongoing hospitalization is for custodial care so inpatient status is acceptable so patient can go to a SNF.
      4. Patient was critically ill and left AMA before the 2^nd^ midnight, but normally would have expected to have stayed longer.
  14. Which is FALSE? ( Circle Correct Answer)
      1. Commercial payors usually are less flexible on inpatient status compared to Medicare and do not allow attending discretion.
      2. Medicare looks at the expected discharge date crossing 2 midnights and there must be active medical care being provided in this estimate by the provider in their documentation.
      3. Commercial payors typically use MCG or Interqual to determine whether a patient meets inpatient status and hospital denials occur when the payor sees this is not met.
      4. A patient cannot be in observations status for days for Medicare.
  15. Provide improved areas of documentation to what is listed below (Fill in Blank):

(Example: Heart failure: Acute CHFpEF)

- - 1. Respiratory Distress (hypoxia on 5 L and tachypnea) ___________________________
    2. Renal insufficiency __________________________
    3. Pneumonia ___________________________
    4. GI blood loss/Anemia ____________________________
  1. What is the purpose of improved clinical documentation (True/False)?
     1. Improves O:E ratio by painting a more accurate clinical acuity and severity of comorbidities _____
     2. Your clinical documentation directly influences how 3rd party organizations interpret the quality of

the care provided. _____

- - 1. Patient healthcare choices from public rankings are based on documentation among other things. _____
    2. Documentation makes patients look sicker than reality to improve hospital profits. _____
    3. By capturing secondary diagnoses with comorbid or morbid conditions (CCs/MCCs), the diagnosis-related group (DRGs) of the principal diagnosis increases which improves quality measures. _____
  1. Identifying Hospital Leaders (Match for each position)

| System Chief Clinical Officer |
| --- |
| Regional Vice President, Acute Care Quality, Patient Safety, and Patient Experience – North Region |
| Chief, Stewardship and Physician Advisor Services – North Region |
| Associate Chief Quality Officer for Ambulatory Care |
| Director – Health Equity Impact Team |
| Evanston Hospital President |
| Medical Director – Care Management |
| Assistant Vice President - Data Analytics |
| Regional Vice President, Cross Care Continuum - North Region |
| Chief Executive Officer |

**POST TEST ONLY ADDITIONAL QUESTIONS:**

1. Please give feedback about **what worked well** in the QI/PS and Physician Advisor curriculum

1. Please give feedback about **what could be improved** in the QI/PS and Physician Advisor curriculum
2. What learning objectives weren’t met or could have been defined better for you?
3. If you could re-structure this rotation (amount of time spent on each activity, length as an elective or have chunks year-long, what you found least useful), what are 1-2 things would you change? What are 1-2 things most helpful that you wish you could have spent more time on during the rotation?

**If you enjoyed this rotation, please spread the word to your co-residents as that’s the only way we can continue providing it.**
